# Pyrazinamide Effects on Cartilage Type II Collagen Amino Acid Composition

**DOI:** 10.1155/2012/781785

**Published:** 2012-05-07

**Authors:** Larysa B. Bondarenko, Valentina M. Kovalenko

**Affiliations:** SI “Institute of Pharmacology and Toxicology” National Academy of Medical Sciences of Ukraine, Eugene Potier 14, 03680 Kyiv, Ukraine

## Abstract

*Introduction*. Current therapeutic regimens with first-line antitubercular agents are associated to a high rate of adverse effects which could cause pronounced changes in collagen's contents and structure. Investigation of these changes is very important for optimization of antitubercular therapy and minimization of treatment-caused harm. The aim of present paper was to investigate potential effect of pyrazinamide on male rats' cartilage type II collagen amino acid composition. *Materials and Methods*. Wistar albino male rats (160–200 g b.w.) were divided into three groups: I—received pyrazinamide *per os* at a dose of 1000 mg/kg b.w./day; II—at a dose of 2000 mg/kg b.w./day, in both groups it was given for 60 days; III—control. After 60 days of the experiment, rats of the experimental (groups I and II) and control groups were sacrificed and the amino acids contents of male rat cartilage type II collagens were determined using amino acid analyzer. *Results and Discussion*. The study of pyrazinamide effects (administered in different doses) on rat cartilage type II collagen amino acid contents demonstrated presence of dose-dependent pyrazinamide-mediated quantitative and qualitative changes in these rat extracellular matrix proteins in comparison with control.

## 1. Introduction

There has been resurgence in tuberculosis worldwide. Approximately 2 billion people have latent infection, 8 million would develop active tuberculosis annually, and 2-3 million would die due to tuberculosis. With this resurgence, cases with extrapulmonary tuberculosis have also shown an increase. Approximately 10-11% of extrapulmonary tuberculosis involves joints and bones, which is approximately 1–3% of all tuberculosis cases. The global prevalence of latent joint and bone tuberculosis is approximately 19–38 million cases [1].

Collagens are major structural proteins of the extracellular matrix, joints, and bones and their correct structure is crucial for the proper functioning of locomotor apparatus. Both tuberculosis per se and its chemotherapy with antitubercular drugs could cause pronounced changes in collagen's contents and structure [1, 2]. Investigation of these changes is very important for improving first-line antitubercular therapy and minimization of its adverse effects.

Previously we have demonstrated putative changes in rat bone and skin type I collagens amino acid contents with using different doses of pyrazinamide [2, 3]. Type II collagen has been classically recognized as the major collagenous component of cartilage. 

The aim of present study was to investigate potential effect of pyrazinamide on male rats cartilage type II collagen amino acid composition.

## 2. Materials and Methods

Cartilage type II collagens were extracted and purified according to Trelstad et al. [4]. All procedures were carried out at 4°C. Cartilages (20 g) were grinded. Extraction of proteoglycans was carried out by 100 mL 2 M MgCl_2_, 0.05 M Tris (pH 7.6) during 3 days. Extract was decanted. Cartilages were washed by distilled water (3 times). Collagen was extracted by 100 mL 0.1 M acetic acid (pH 2.5) with pepsin. Pepsin (20 mg/g of tissue) was added into this solution and mixtures were left for 3 days in refrigerator at 4°C. After that for pepsin inactivation pH in each mixture was neutralized by addition of powdered crystalline Tris (to pH 7.6). Solutions were centrifuged: 35000 g, 40 min, 4°C. Pellets were discarded and supernatants were used for further collagen types fractionation. Fractionation of pure type II collagens was carried out by growing concentrations of NaCl according to method [4]. Protein fraction from pellet which was formed at 4.4 M NaCl concentration contained type II collagen. Fractions were separated by centrifugation (65000 g, 60 min, at 4°C). Obtained pellets were recrystallized (3 times) by dialysis (against 15% KCl in 0.02 M NaHPO_4_ at 4°C) and centrifugation (65000 g, 60 min, at 4°C) [5]. Collagen preparations purity was controlled electrophoretically [6].

Collagen fractions were hydrolyzed: 24 h, 6 N HCl, 105°C [7]. Their amino acid compositions were analyzed by ion exchange chromatography on the amino acid analyzer AAA-881 (Czech Republic).

In statistical processing of experimental data mean of corresponding parameter (for each animal) was used as independent variable. The obtained data were calculated by one-way analysis of variance (ANOVA). Data were compared using Tukey test. Differences were considered to be statistically significant at *P* < 0.05.

## 3. Results and Discussion

 Changes in male rat cartilage type II collagen amino acid contents induced by pyrazinamide were profound as compared to control (Table 1). Statistically significant changes were registered in cartilage collagen with pyrazinamide administration at dose 1000 mg/kg for 6 amino acids and at dose 2000 mg/kg for 6 amino acids.

Cartilage type II collagen of male rats with pyrazinamide at dose 1000 mg/kg contains lower contents of alanine (−12.3%) and isoleucine (−37.8%) simultaneously with higher contents of serine (+46.5%), glutamic acid (+31.0%), and leucine (+46.6%). Collagen of rats with pyrazinamide at dose 2000 mg/kg contains lower contents of arginine (−25.9%) and alanine (−31.7%) simultaneously with higher contents of serine (+65.6%), valine (+32.7%), and leucine (+40.6%). For the majority of amino acids pyrazinamide effects were dose dependent.

Our experiments demonstrated presence of qualitative changes in male rats' cartilage type II collagens with pyrazinamide (in comparison with norm) (Table 1). With pyrazinamide administration possibly could be formed cartilage type II collagen molecules with changed surface charge (changes in number of arginine, serine, and glutamic acid residues), rigidity (changes in quantity of alanine, valine, isoleucine residues), number of specific loci responsible for cell adhesion, interaction with chaperons, and procollagen processing to collagen (changes in arginine residues) [8–14]. Such collagen molecules changes could hence affect the properties of connective tissues, mineralisation processes, and calcium metabolism.

Comparative analysis of present results with our previous data on skin and bone type I collagens demonstrated analogous character of changes in regard to contents of serine, glutamic acid, alanine, valine, and leucine residues [2, 3]. This could be the evidence of existence some general mechanisms of pyrazinamide effects on collagen's contents and structure. Moreover, having compared these data with our previous experiments, we found out analogous character of these changes in regard to changes of free serine, glutamic acid, alanine, valine, and leucine contents in liver, kidney, lung and spleen pools with different doses of pyrazinamide [8]. Thus adverse effects of pyrazinamide (this widely used antitubercular drug) are much more serious and more profound than it was considered earlier. Among this pyrazinamide treatment could cause qualitative changes in nucleic acid molecules, change their length, and structure [9].

We can suppose that such changes could be caused by pyrazinamide via its influence on nucleic acids (coding information for this proteins synthesis) as it was mentioned previously [2, 9, 10]. In our previous experiments we demonstrated epigenetic changes induced by pyrazinamide treatment, pyrazinamide-mediated alterations in male rats DNA fragmentation processes, bone type I collagen amino acid composition, spermatogenesis indices, reproductive capability, and posterity antenatal and postnatal development. Besides these, on changes in collagen metabolism and structure pathologic changes in amino acid metabolism could also be affected [8]. And at last, due to collagen genes polymorphism [11–14], collagen structures contain in norm 4 different *α*-chains of the same type in different concentrations. Pathology [14] changed concentrations in which these 4 different *α*-chains of the same type of collagen are present in connective tissue structures. Possibly pyrazinamide-caused disturbances in amino acid compositions in our experiments could be a result of such changes in transcription rates of genes coding different *α*-chains from the same type collagen superfamily as it was previously demonstrated for other pathology [14].

## Figures and Tables

**Table 1:** Male rats cartilage type II collagen amino acid contents in control and with pyrazinamide administration at doses 1000 mg/kg and 2000 mg/kg of body weight (M ± m, *n* = 5, residues/1000 residues).

Amino acid	Control (norm)	Pyrazinamide 1000 mg/kg	Pyrazinamide 2000 mg/kg
Hydroxylysine	5.60 ± 1.20	4.20 ± 0.80	3.50 ± 0.40
Lysine	29.9 ± 1.80	31.80 ± 2.70	33.30 ± 2.50
Histidine	8.30 ± 1.20	7.10 ± 1.30	5.60 ± 0.30
Arginine	53.83 ± 3.35	53.00 ± 5.80	39.90 ± 3.00^∗#^
Hydroxyproline	95.80 ± 2.70	91.70 ± 4.70	85.90 ± 2.90
Aspartic acid	53.50 ± 5.10	62.30 ± 6.40	72.10 ± 10.60
Threonine	29.80 ± 6.60	29.20 ± 1.30	33.70 ± 2.10
Serine	34.00 ± 1.98	49.80 ± 1.60*	56.30 ± 2.30^∗#^
Glutamic acid	88.10 ± 3.47	115.40 ± 6.30*	110.80 ± 12.20*
Proline	91.70 ± 1.60	91.20 ± 4.10	86.10 ± 3.70
Glycine	310.20 ± 7.70	282.10 ± 9.90	294.00 ± 6.40
Alanine	105.62 ± 2.78	92.60 ± 1.80*	72.10 ± 5.50^∗#^
Valine	24.80 ± 1.89	16.70 ± 3.60*	32.90 ± 1.57^∗#^
Methionine	7.60 ± 1.50	3.80 ± 1.00	2.70 ± 0.70
Isoleucine	13.35 ± 0.79	8.30 ± 0.80*	10.60 ± 1.80
Leucine	23.43 ± 1.85	34.30 ± 2.00*	32.90 ± 1.00*
Tyrosine	7.30 ± 0.80	6.80 ± 0.30	7.50 ± 0.50
Phenylalanine	18.80 ± 0.80	20.80 ± 2.30	20.60 ± 2.10

M ± m: mean ± mean standard error.

*P < 0.05 statistically significant in comparison with control.

^#^ *P* < 0.05 statistically significant pyrazinamide, 1000 mg/kg group versus pyrazinamide, 2000 mg/kg group.
